# The Facial Affective Scale as a Predictor for Pain Unpleasantness When Children Undergo Immunizations

**DOI:** 10.1155/2014/628198

**Published:** 2014-03-05

**Authors:** Stefan Nilsson, Berit Finnström, Evalotte Mörelius, Maria Forsner

**Affiliations:** ^1^School of Health Sciences, University of Borås, 501 90 Borås, Sweden; ^2^Institute of Health and Care Sciences, University of Gothenburg, P.O. Box 457, 405 30 Gothenburg, Sweden; ^3^Department of Nursing, Health and Culture, University West, 461 86 Trollhättan, Sweden; ^4^Division of Activity, Health and Care, Department of Social and Welfare Studies, Linköping University, 601 74 Norrköping, Sweden; ^5^School of Health, Pedagogy and Social Studies, Dalarna University, 791 88 Falun, Sweden

## Abstract

Needle fear is a common problem in children undergoing immunization. To ensure that the individual child's needs are met during a painful procedure it would be beneficial to be able to predict whether there is a need for extra support. The self-reporting instrument facial affective scale (FAS) could have potential for this purpose. The aim of this study was to evaluate whether the FAS can predict pain unpleasantness in girls undergoing immunization. Girls, aged 11-12 years, reported their expected pain unpleasantness on the FAS at least two weeks before and then experienced pain unpleasantness immediately before each vaccination. The experienced pain unpleasantness during the vaccination was also reported immediately after each immunization. The level of anxiety was similarly assessed during each vaccination and supplemented with stress measures in relation to the procedure in order to assess and evaluate concurrent validity. The results show that the FAS is valid to predict pain unpleasantness in 11-12-year-old girls who undergo immunizations and that it has the potential to be a feasible instrument to identify children who are in need of extra support to cope with immunization. In conclusion, the FAS measurement can facilitate caring interventions.

## 1. Background

Immunizations are common procedures in the life of most children [1], preventing diseases that inflict disability or death. The human papillomavirus (HPV) vaccination aims to protect against the development of cancer caused by HPV. Administration of the vaccination is recommended before sexual debut in order to maximize the effectiveness of the immunization [2]. However, immunizations are the most common iatrogenic intervention posing a risk of pain for children [3], and the children's cognitive as well as emotional needs ought to be taken into consideration when planning for caring interventions related to immunizations. According to Galvin and Todres [4], caring can be divided into three domains: the head, the hand, and the heart. The head involves existing knowledge of the context; the hand demonstrates the nurse's practical processes of caring; and the heart represents the nurse's ability to meet individual needs.

Engaging the heart in caring means taking individuals' health-related experiences into consideration [4]. Children report that they can feel threatened when in contact with the health care system, and needle-related procedures in particular make them afraid [5]. Taddio et al. [6] found that 63% of healthy children aged 6 to 17 years reported having a fear of needles. When afraid, children ask for extra support to endure medical procedures [5] and consequently require extra caring interventions. However, providing extra caring interventions for every child could be considered too time consuming and, furthermore, undermine the children's own coping skills. Consequently, identifying those who need extra support would be advantageous.

According to Galvin and Todres' [4] knowledge, it is important to consider the context of the pain. The neuromatrix theory includes emotional and cognitive aspects of the way pain signals are interpreted in the central nervous system [7]. Every previous psychological incident in life creates a neurosignature in the brain, and this signature is recalled for the individual when a similar context recurs [8]. This knowledge encourages nurses to examine past experiences of similar contexts, as earlier pain memories may affect the child in a negative way [9]. Earlier pain experiences of immunizations may affect the child's coping, and these experiences have to be highlighted when planning for and performing a procedure [10]. Previous experiences of anxiety explain the child's pain memory and his or her reaction the next time procedural pain occurs. This highlights the need for nurses to explore children's anxiety related to needle procedures, as this plays an important role in the child's development of pain memories in connection with immunizations [11].

Carter [12] emphasizes that nurses need to listen to and notice the things children perceive as important and engage with children's pain on their own terms. This means being prepared to care for children with the heart, mind, and self, as well as using all the tools and technology available. This is in accordance with Galvin and Todres [4] emphasis on the head, the hand, and the heart as equally important domains of caring. Consequently, nurses have to be aware of the fact that some children need extra support, that is, using the heart, relating to the way pain memories can be developed, that is, using the head. To be able to meet the individual child's needs, it may be beneficial to predict the need for extra support, that is, using both the head and the hand, facilitating individualized caring interventions, that is, using the heart, for each child undergoing needle-related procedures, such as immunizations.

Galvin and Todres [4] highlight that a nurse needs to use the heart to conduct empathic responding. However, it should be noted that confirming the child's emotions using pain-related words may influence the processing of the central nervous system associated with the cognitive dimension of pain [13]. For this reason, strategies that reduce children's emotional response in conjunction with needle-related procedures are of utmost importance [14].

**Figure 1:**
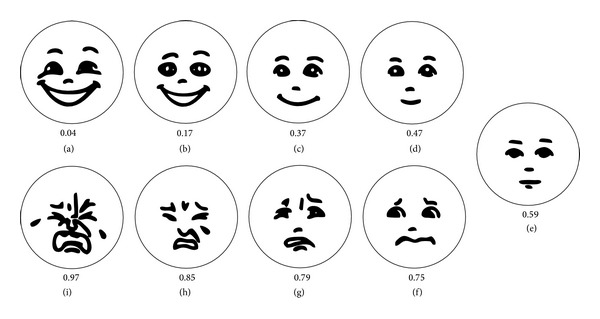
The facial affective scale.

Patient-centred care means involving the patient in decisions about their own health issues and is regarded as important for good quality [15]. The UN Convention on the Rights of the Child stresses the need for society to enable children to express their own views on all matters affecting themselves [16]. Nurses are therefore expected to take the child's perspective and establish whether children experience pain unpleasantness in conjunction with immunizations. Self-reports tend to take the child's perspective, and they should be the first choice. Self-reports in these circumstances are valuable for capturing children's pain unpleasantness, but the level of interpretation needed to understand self-reports can vary. However, the design of the instrument can facilitate this understanding [17]. The facial affective scale (FAS) developed by McGrath et al. [18] gives children from the age of five an opportunity to report their affective reactions related to pain by marking one of nine faces presented in an ordered sequence from “happiest feeling possible” (0.04) to “saddest feeling possible” (0.97) (Figure 1) [18]. An advantage of the FAS is that this scale has demonstrated a discrepancy between pain unpleasantness and pain intensity, consequently catching the emotional component of pain [19]. The assessment of the individual child's FAS score is a method that facilitates the use of the child's perspective, as it will encourage a discussion of pain unpleasantness between the child and the nurse on the child's level of knowledge and experience. Hence, the child's previous experience and future expectation are considered [17]. The FAS score can help nurses to find children in need of extra support [18]. This knowledge may facilitate these children learning coping strategies, such as distraction, before they undergo the vaccination. Distraction has been shown to reduce procedural pain [14].

Additionally it has been shown that pictures, such as faces, facilitate children being able to understand and communicate the meaning of pain unpleasantness in contrast to pain intensity [19]. However, whether the FAS can predict children's experience of pain unpleasantness is unknown.

## 2. Objectives

The aim of this study was to evaluate whether the FAS can predict pain unpleasantness in girls undergoing immunization.

## 3. Methods

### 3.1. Participants

The participants in the study were females aged 11 to 12 years from three different schools located in western Sweden who were fluent in the Swedish language. The participants were offered vaccinations against HPV. Immunization against HPV is part of the Swedish vaccination programme for girls and is given on three occasions.

### 3.2. Instruments

#### 3.2.1. Pain Unpleasantness

The primary outcome in this study is whether the FAS scores of expected pain unpleasantness predict the FAS scores of experienced pain unpleasantness. The FAS scores assess a child's emotional or affective reaction to a pain experience and range from “happiest feeling possible” to “saddest feeling possible.” It only takes approximately half a minute to administer and assess the FAS score [20].

#### 3.2.2. Anxiety

Anxiety is described as a complex combination of fear, apprehension, and worry. Anxiety scores can be assessed by a visual analogue scale (VAS-A) with the anchor's “no anxiety” and “the worst known anxiety.” The VAS-A has shown validity in assessing perioperative anxiety in children aged 7–16 years and has shown significant correlations with the STAIY (State-Trait Anxiety Inventory for Youth) [21]. In the present study, the VAS-A was used to assess and evaluate the concurrent validity of the FAS.

#### 3.2.3. Stress

Stress affects the body and the brain in many ways and appears when a person experiences a threat. The verbal rating scale for stress (VRSS) reports the recent experience of stress on a verbal rating scale (0–5). A test of VRSS showed no statistical evidence for systematic disagreement or random disagreement, which indicates that it is a valid and reliable scale [22]. In the present study, VRSS was used to assess and evaluate the concurrent validity of the FAS.

### 3.3. Data Collection

**Figure 2:**
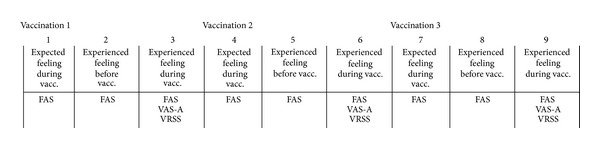
Measurement points.

Data were collected between September 2012 and September 2013. Each child reported FAS scores three times in conjunction with each vaccination, that is, a total of nine times (Figure 2). Before each vaccination all participants received a letter sent to their homes, and they were asked to report their expected feelings prior to the vaccination. The level of pain unpleasantness was reported on the FAS, conducted approximately two weeks before the first vaccination. The FAS scoring before the subsequent vaccination occasions was conducted approximately two weeks after the previous vaccination. After further two weeks, those who did not return their self-report received a reminder. This procedure was repeated after each vaccination. The girls assessed their expected pain unpleasantness on the FAS at least two weeks before the vaccination to minimize influence between the different measurement points.

The participants also reported their experienced pain unpleasantness in relation to each of the three vaccinations, just before and during the immunization. The scores during the immunizations were collected immediately afterwards.

To further test the concurrent validity of the FAS, two other instruments were used in conjunction with each immunization. The girls reported their anxiety on a VAS-A, and the scores during the immunizations were collected immediately afterwards [21]. In addition, the girls reported their VRSS score after each immunization; this score reflected the experience of stress during the immunization [22].

### 3.4. Data Analysis

The statistics were calculated using IBM SPSS Statistics for Windows, version 21. The FAS has been validated in earlier studies using parametric statistics [18], which led to the selection of equal statistics in this study. The study population was deemed sufficient compared with Heden et al., who calculated the clinical significance using parametric statistics [23].

The FAS scores before each immunization were compared with the FAS scores during each immunization using linear regression analysis. The linear regression analysis was repeated for each immunization, that is, three times. The dependent variable was the output, that is, the experienced pain unpleasantness, and the independent variable was the expected pain unpleasantness. A beta score close to 1 indicates a strong connection between independent and dependent variables.

The comparison between experienced pain unpleasantness (FAS scores) before the immunizations and the experienced pain unpleasantness (FAS scores) during the immunizations was tested by a paired *t*-test.

The concurrent validity was tested by a Pearson correlation test that compared the FAS scores and the VRSS scores as well as the FAS scores and the VAS-A scores during the immunizations. A correlation coefficient close to 1 indicates a strong correlation [24].

### 3.5. Ethical Considerations

Guidelines for research involving human subjects were followed. Written information was provided to both children and parents, and verbal information was given in school. Voluntariness as well as the right to withdraw from the study at any time without any explanation and consequences was highlighted. The benefit of the study was considered greater than the risks. If the children agreed to participate, the parents were asked for written consent. All participants received a cinema ticket. The study was approved by the regional ethical review board (Dnr: 466-12).

## 4. Results

### 4.1. Study Participants

Two invited girls declined to participate and one girl dropped out of the offered immunization and consequently from this study. In the end, 37 study participants, aged 11 to 12 years, participated in this study. Some of the study participants declined to report their expected pain unpleasantness on the FAS; 31 study participants (84%) reported their expected pain unpleasantness before the first immunization, 28 study participants (76%) before the second immunization, and finally 32 study participants (86%) before the third immunization. All study participants assessed their pain unpleasantness before and during each vaccination and anxiety and stress during each vaccination.

### 4.2. Expected Pain Unpleasantness

Most study participants reported a moderate level of expected pain unpleasantness on the FAS (mean 0.56) before all three immunizations (Table 1). The level of expected pain unpleasantness differed between the study participants (Table 2).

### 4.3. Experienced Pain Unpleasantness

The study participants reported their pain unpleasantness just before the first (mean 0.64), second (mean 0.58), and third vaccinations (mean 0.52).

Thirty-seven study participants reported their experienced pain unpleasantness, anxiety, and stress during each immunization (Table 3).

### 4.4. Correlations between Expected and Experienced Pain Unpleasantness

The study participants also reported moderate experienced pain unpleasantness on the FAS during all three immunizations. However, the FAS scores did not increase significantly from directly before to immediately after, which reflected the feelings during the first (*P* = 0.05), second (*P* = 0.06), or third (*P* = 0.56) occasions of immunization.

The comparison of the expected pain unpleasantness (FAS scores) before the first vaccination and the experienced pain unpleasantness (FAS scores) during the first vaccination showed beta 0.59 (*P* = 0.001), the expected pain unpleasantness (FAS scores) before the second vaccination, and the experienced pain unpleasantness (FAS scores) during the second vaccination showed beta 0.75 (*P* < 0.001), and finally the expected pain unpleasantness (FAS scores) before the third vaccination and the experienced pain unpleasantness (FAS scores) during the third vaccination showed beta 0.64 (*P* < 0.001).

The Pearson correlation test showed significant correlations between the FAS, VAS-A, and VRSS during all three of the immunizations (Table 4).

## 5. Discussion

The self-reporting instrument, FAS, acceptably predicted adolescent study participants' level of pain unpleasantness when undergoing immunizations. Since needle-related fear has been found to be as common as in 63% of children and youth [6], nurses are challenged to reach those requiring extra support. The finding of this study brings hope for achievements in the future for nursing care for needle-related procedures such as immunizations. Following Galvin and Todres [4], skills in the practical processes of caring as well as knowledge about the context are required to enable nurses to meet individual needs. In this case, knowledge about how to predict pain unpleasantness during HPV vaccination enables nurses to plan for caring interventions and strive to reduce the potential negative experience. Additionally, the study revealed that FAS showed a good to high level of concurrent validity with both the VAS-A and VRSS measures, suggesting that the FAS captures a global rating of the child's emotions rather than specific stress or anxiety. This is in accordance with Perrott et al. [25] defining the FAS as an instrument describing children's emotions globally and ranging from a happy to a sad condition.

The study participants' FAS scores did not significantly change over time, making it possible to use this instrument to predict pain unpleasantness. The FAS may then be used for screening to identify children who will experience immunization unpleasantness. This facilitates providing these children with extra support and can perhaps help them to cope better with the procedure during immunizations. Moreover, if nurses use a validated assessment scale to find children who need extra support it will minimize the risk of nurses using their own preunderstanding of pain instead of the children's perspectives [17], which is in accordance with the UN Convention on the Rights of the Child [16].

Despite the fact that some study participants did not expect or experience pain unpleasantness, the number scoring substantial pain unpleasantness (FAS score ≥ 0.75) should not be ignored. Similarly, an estimated one-sixth of children aged 12 assessed high FAS scores both before and during a needle-related procedure [26]. The results in the current study also showed mean values of the FAS scores that pointed out pain unpleasantness as an issue to highlight. If the FAS score shows that a child is in need of extra support, this support can be implemented before the pain unpleasantness appears.

It is an advantage if an instrument is easy to use and can actually predict pain unpleasantness. This study showed that the FAS instrument has the potential to meet this requirement. While the FAS discriminates pain unpleasantness from pain intensity [20] and additionally assesses pain unpleasantness in conjunction with procedural pain [27], it has not previously been used to predict this reaction. It is important that the child is able to gain control in this situation [28]. It may be difficult to learn new strategies in direct conjunction with the procedure causing pain unpleasantness.

Children who undergo immunizations can feel uncomfortable, that is, experience a fear of needles. However, not all children are afraid of immunizations. Forsner et al. found that children's emotional experiences when undergoing a venepuncture varied greatly, and some of the children's narratives even suggested that they “love shots” [26]. Those children who can easily manage the situation are perhaps not candidates for extra caring interventions. Nevertheless, the consequences of missing a child who really is in need of extra support are significant, involving, for instance, unnecessary suffering for the child and furthermore requiring time-consuming interventions in order to help the child to cope with upcoming situations concerning needle-related procedures [9].

Carter [12] stresses that besides nursing skills and technique, nurses need to be empathetic in order to understand children's feelings. Forsner et al. [5] similarly emphasize that caring means letting the fearing child's emotion into the heart in order to make caring creativity available. However, it should not be forgotten that using pain-related words in communication with the child may have a negative influence on the experienced pain [13]. Beyond using the heart in caring, Galvin and Todres [4] emphasize that nurses need to use the head and the hand to offer children optimal caring [4]. The ability to screen and identify children who need extra support makes it easier for nurses to fulfil caring activity and prevent unnecessary suffering. The current study showed that the FAS could be used as a screening method and, fortunately, does not require that much time. The FAS is thereby a way for nurses to use both their head and their heart and to respond to the individual child's own experience. Nurses need to identify those children who experience pain unpleasantness and offer them caring interventions, which may prevent further negative pain memories [29].

Nurses administer immunizations to children and thereby contribute to child health, but, unfortunately, some children are afraid of needle procedures [26]. This fact provides good reason for nurses to help children who predict a high level of pain unpleasantness. To achieve this, nurses need knowledge of interventions that reduce pain unpleasantness, according to Galvin and Todres [4]: using their heads. This means that nurses gain knowledge that they can apply in their caring of children undergoing immunizations. Furthermore, they need to actually use this knowledge and offer individualized caring intervention, that is, use their hands [4]. There are several evidence-based interventions to offer children who are in need of extra support during immunization [14]. An important start to prevent children's pain unpleasantness is to inform them about what will happen. The knowledge about what will happen gives the child a sense of control, and this can often be an important key to success when children undergo needle-related procedures [28]. Additionally, combining local anaesthesia with both sensory and procedural information was found to reduce distress reactions in young children undergoing a needle procedure [29]. Consequently, preparations before immunizations are important and facilitate children having influence on their own health care [16]. Furthermore, offering children distraction, such as guided imagery, has been found to be a pain-reducing intervention [14]. Music medicine [30] and video games [27] have also been found to help children cope with procedural pain. However, it is important to ask each child about his or her desire to cope with the situation. Not all distractions suit all individuals [31]. Some children benefit more from watching the needle-related situation than from being distracted, and children's coping styles have consequently been categorized as either “attenders” or “distracters.” However, when given distraction training and offered the option to choose at follow-up, both “distracters” and “attenders” preferred to use distraction [32].

Warning or sympathizing using language that refers to negative experiences may not facilitate children feeling better [33], but there is value in focusing on pain unpleasantness in children. The ability to discuss with children how they will manage the pain unpleasantness during the vaccination, that is, taking control, has been successful in conjunction with procedural pain [34]. The nurse ought to discuss and decide in consultation with the child how the practical processes should be carried out in conjunction with the immunizations. In this way, the nurse listens to the child and takes the child's perspective [17], as prescribed in the UN Convention on the Rights of the Child [16]. The assessment with the FAS takes hardly any time, and future research could look at the feasibility of incorporating this as a standard instrument in clinics.

One limitation of this study is the small population. The study ought therefore to be repeated with a larger population. On the other hand, the study design was strengthened by the fact that there were no dropouts during the immunizations and, in addition, it permitted each child to repeat the measurement on the FAS up to nine times. The fact that only girls participated in the study could also be considered a weakness, but, to date, only girls have received HPV immunizations in Sweden. It would in any case be valuable to repeat the study design with boys. Finally, since the findings are limited to young adolescents, further studies should focus on whether children in other age groups are able to predict own pain unpleasantness on the FAS.

## 6. Conclusion

The FAS is valid for predicting, as well as measuring, pain unpleasantness in 11-12-year-old girls who undergo immunizations. This instrument consequently has the potential to identify children who are in need of extra support to cope with the situation, facilitating individualized prevention and caring interventions for children undergoing immunizations.

## Figures and Tables

**Table 1:** Expected level of pain unpleasantness using the FAS.

	*n*	Min.	Max.	Mean	SD
Expected pain unpleasantness 1	31	0.17	0.75	0.56	0.20
Expected pain unpleasantness 2	28	0.04	0.85	0.61	0.24
Expected pain unpleasantness 3	32	0.04	0.85	0.61	0.22

**Table 2:** The number and proportions of study participants who did or did not feel pain unpleasantness.

	Vaccination 1		Vaccination 2		Vaccination 3	
	*n*		*n*		*n*	
Expected FAS						
FAS score ≥0.75	14/31	45%	15/28	54%	19/32	59%
FAS score ≤0.47	13/31	42%	7/28	25%	11/32	34%
Experienced FAS						
FAS score ≥0.75	17/37	46%	21/37	57%	11/37	30%
FAS score ≤0.47	11/37	30%	9/37	24%	16/37	43%

**Table 3:** Descriptive statistics.

	Min.	Max.	Mean	SD
First immunization				
Experienced FAS	0.04	0.85	0.59	0.22
Experienced VAS-A	0	9	4.02	2.51
Experienced VRSS	0	4	1.56	1.03
Second immunization				
Experienced FAS	0.04	0.85	0.63	0.20
Experienced VAS-A	0	8.5	3.78	2.36
Experienced VRSS	0	4	1.51	1.15
Third immunization				
Experienced FAS	0.04	0.85	0.54	0.21
Experienced VAS-A	0	6.6	2.63	2.02
Experienced VRSS	0	3	1.19	0.94

**Table 4:** The Pearson correlation test.

Immunization	1	2	3
	FAS	FAS	FAS
VAS-A	*r* = 0.75 (*P* < 0.05)	*r* = 0.69 (*P* < 0.05)	*r* = 0.73 (*P* < 0.05)
VRSS	*r* = 0.70 (*P* < 0.05)	*r* = 0.66 (*P* < 0.05)	*r* = 0.76 (*P* < 0.05)
